# Supplementary material for: Induction of Brain Insulin Resistance and Alzheimer’s Molecular Changes by Western Diet
Source: Int J Mol Sci. 2022 Apr 25;23(9):4744. doi: 10.3390/ijms23094744 (PMC9102094; doi:10.3390/ijms23094744)
Supplement: Supplementary file 1 [file ijms-23-04744-s001.zip › ijms-1664808-Supplementary Materials.pdf]

Supplementary Materials

Induction of brain insulin resistance and Alzheimer's molecular changes by Western diet

Anna Mietelska-Porowska[1] †, Justyna Domańska[1] †, Andrew Want[1], Angelika Więckowska-Gacek[1], Dominik Chutorański[1], Maciej Koperski[1], Urszula Wojda[1,*]

[1] Nencki Institute of Experimental Biology Polish Academy of Sciences, Warsaw, Poland Laboratory of Preclinical Testing of Higher Standard;

* Correspondence: u.wojda@nencki.edu.pl; Tel.: +48 225892578, UW

† These authors contributed equally to this work.

Figure S1: WD-derived insulin signaling impairment on AD biomarkers, analyzed for extreme values for each individual in all experimental and age groups (paragraph 2.3).

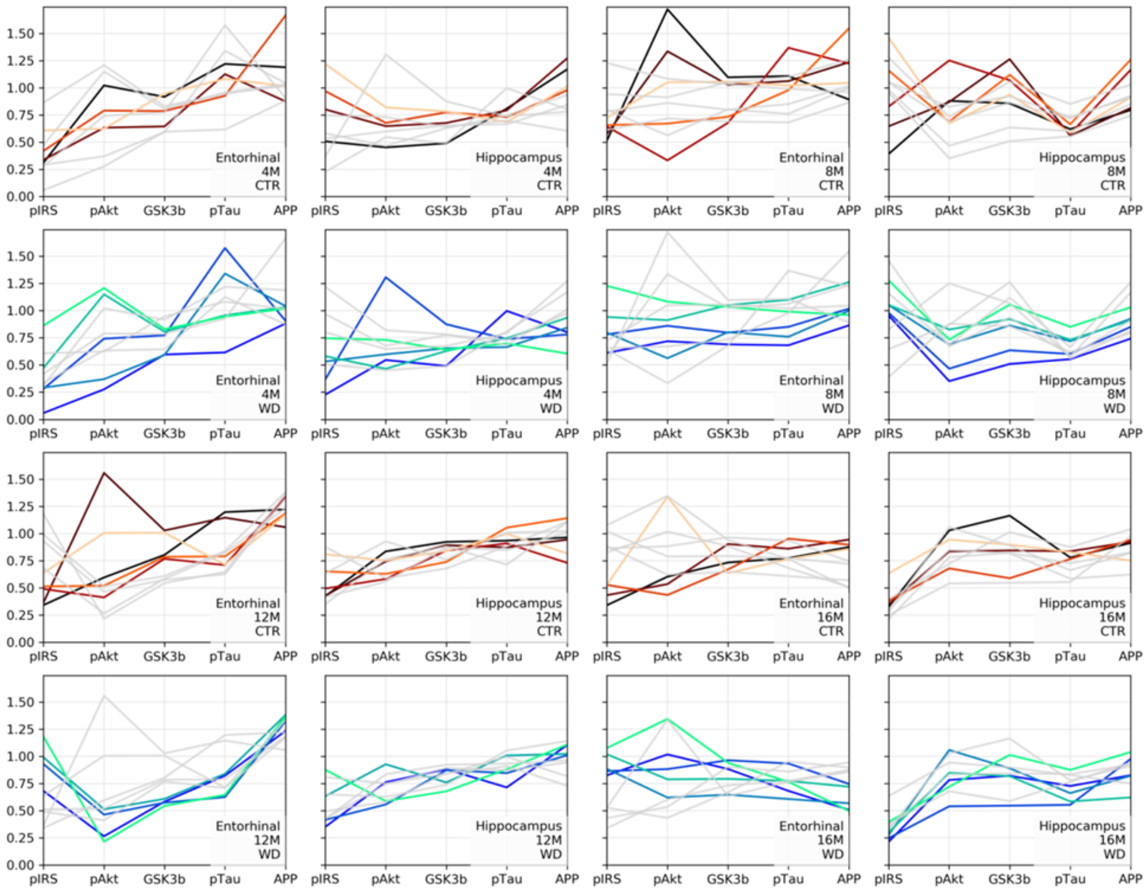

Table S1: Completed statistical data – analysis of peripheral markers in C57BL/6 mice (paragraph 2.1)

Figure number: Analyzed parameter	Age groups	Statistical test	Significance	p-Value	Value of F (DFn, DFd)
Fig.1. Glucose	ALL	2way ANOVA	Interaction: ns<br>Age: ns<br>Diet: **	Interaction: 0.2284<br>Age: 0.1503<br>Diet: 0.0077	Interaction: $F_{(3,28)}$ = 1.530<br>Age: $F_{(3,28)}$ = 1.914<br>Diet: $F_{(1,28)}$ = 8.255
Fig.1. Glucose	4M vs. 16M WD WD	Tukey's post-hoc	*	0.0404	-
Fig.1. Insulin	ALL	2way ANOVA	Interaction: ns<br>Age: ns<br>Diet: **	Interaction: 0.3169<br>Age: 0.2901<br>Diet: 0.0094	Interaction: $F_{(3,28)}$ = 1.231<br>Age: $F_{(3,28)}$ = 1.312<br>Diet: $F_{(1,28)}$ = 7.787
Fig.1. HOMA-IR	ALL	2way ANOVA	Interaction: ns<br>Age: ns<br>Diet:**	Interaction: 0.1056<br>Age: 0.2196<br>Diet:0.0026	Interaction: $F_{(3,28)}$ = 2.240<br>Age: $F_{(3,28)}$ = 1.567<br>Diet: $F_{(1,28)}$ = 10.98
Fig.2. p-IRS-1 (Ser616) Entorhinal cortex	ALL	2way ANOVA	Interaction: *<br>Age: **<br>Diet: ****	Interaction: 0.0165<br>Age: 0.0010<br>Diet: <0.0001	Interaction: $F_{(3,29)}$ = 4.023<br>Age: $F_{(3,29)}$ = 7.104<br>Diet: $F_{(1,29)}$ = 25.18
Fig.2. p-IRS-1 (Ser616) Entorhinal cortex	4M vs. 8M WD WD	Tukey's post-hoc	**	0.0042	-
Fig.2. p-IRS-1 (Ser616) Entorhinal cortex	4M vs. 12M WD WD	Tukey's post-hoc	**	0.0016	-
Fig.2. p-IRS-1 (Ser616) Entorhinal cortex	4M vs. 16M WD WD	Tukey's post-hoc	***	0.0010	-
Fig.2. p-IRS-1 (Ser616) Entorhinal cortex	12M vs. 12M CTR WD	Tukey's post-hoc	**	0.0084	-
Fig.2. p-IRS-1 (Ser616) Entorhinal cortex	16M vs. 16M CTR WD	Tukey's post-hoc	**	0.0081	-
Fig.2. p-Akt (Ser473) Entorhinal cortex	ALL	2way ANOVA	Interaction: ns<br>Age: ns<br>Diet: ns	Interaction: 0.3047<br>Age: 0.2769<br>Diet: 0.3506	Interaction: $F_{(3,29)}$ = 1.265<br>Age: $F_{(3,29)}$ = 1.352<br>Diet: $F_{(1,29)}$ = 0.899

*p<0.05, **p<0.01

Table S2: Completed statistical data – analysis of brain markers in C57BL/6 mice (paragraph 2.2)

Figure number: Analyzed parameter	Age groups	Statistical test	Significance	p-Value	'alue of F (DFn, DFd)
Fig.2:<br>p-GSK-3β (Ser9)<br>Entorhinal cortex	ALL	2way ANOVA	Interaction: *<br>Age: ns<br>Diet: ns	Interaction:0.0297<br>Age: 0.0705<br>Diet: 0.0733	Interaction: $F_{(3,29)}$ = 3.439<br>Age: $F_{(3,29)}$ = 2.608<br>Diet: $F_{(1,29)}$ = 3.454
Fig.2.<br>p-GSK-3β (Ser9)<br>Entorhinal cortex	12M vs. 12M<br>CTR WD	Tukey's post-hoc	*	0.0491	-
Fig.2.<br>p-Tau (Thr231)<br>Entorhinal cortex	ALL	2way ANOVA	Interaction: ns<br>Age: *<br>Diet: ns	Interaction: 0.6246<br>Age: 0.0123<br>Diet: 0.0726	Interaction: $F_{(3,29)}$ = 0.5931<br>Age: $F_{(3,29)}$ = 4.324<br>Diet: $F_{(1,29)}$ = 3.471
Fig.4.<br>p-IRS-1 (Ser616)<br>Hippocampus	ALL	2way ANOVA	Interaction: ns<br>Age: ****<br>Diet: ns	Interaction: 0.0926<br>Age: <0.0001<br>Diet: 0.2727	Interaction: $F_{(3,29)}$ = 2.354<br>Age: $F_{(3,29)}$ = 11.97<br>Diet: $F_{(1,29)}$ = 1.250
Fig.3.<br>p-IRS-1 (Ser616)<br>Hippocampus	4M vs. 8M<br>WD WD	Tukey's post-hoc	*	0.0108	-
Fig.3.<br>p-IRS-1 (Ser616)<br>Hippocampus	8M vs. 16M<br>WD WD	Tukey's post-hoc	***	0.0003	-
Fig.3.<br>p-Akt (Ser473)<br>Hippocampus	ALL	2way ANOVA	Interaction: ns<br>Age: ns<br>Diet: ns	Interaction: 0.3352<br>Age: 0.4925<br>Diet: 0.3518	Interaction: $F_{(3,29)}$ = 1.178<br>Age: $F_{(3,29)}$ = 0.822<br>Diet: $F_{(1,29)}$ = 0.895
Fig.3.<br>p-GSK-3β (Ser9)<br>Hippocampus	ALL	2way ANOVA	Interaction: ns<br>Age: *<br>Diet: ns	Interaction: 0.3954<br>Age: 0.0170<br>Diet: 0.0871	Interaction: $F_{(3,29)}$ = 1.026<br>Age: $F_{(3,29)}$ = 3.993<br>Diet: $F_{(1,29)}$ = 3.137
Fig.3.<br>p-GSK-3β (Ser9)<br>Hippocampus	4M vs. 8M<br>CTR CTR	Tukey's post-hoc	*	0.0372	-
Fig.3.<br>p-Tau (Thr231)<br>Hippocampus	ALL	2way ANOVA	Interaction: ns<br>Age: ****<br>Diet: ns	Interaction: 0.0936<br>Age: <0.0001<br>Diet: 0.4117	Interaction: $F_{(3,29)}$ = 2.344<br>Age: $F_{(3,29)}$ = 11.47<br>Diet: $F_{(1,29)}$ = 0.694
Fig.3.<br>p-Tau (Thr231)<br>Hippocampus	8M vs. 12M<br>CTR CTR	Tukey's post-hoc	****	<0.0001	-

*p<0.05, ***p<0.001, ****p<0.0001

Table S3: Completed statistical data – analysis of brain markers in C57BL/6 mice (paragraph 2.2)

Figure number: Analyzed parameter	Age groups	Statistical test	Significance	p-Value	Value of F (DFn, DFd)
Fig.4. APP Entorhinal cortex	ALL	2way ANOVA	Interaction: ns<br>Age: ****<br>Diet: *	Interaction: 0.0687<br>Age: <0.0001<br>Diet: 0.0234	Interaction: $F_{(3,29)}$ = 2.634<br>Age: $F_{(3,29)}$ = 14.49<br>Diet: $F_{(1,29)}$ = 5.727
Fig.4. APP Entorhinal cortex	4M WD vs. 16M WD	Tukey's post-hoc	*	0.0314	-
Fig.4. APP Entorhinal cortex	8M WD vs. 16M WD	Tukey's post-hoc	*	0.0113	-
Fig.4. APP Entorhinal cortex	12M WD vs. 16M WD	Tukey's post-hoc	****	<0.0001	-
Fig.4. APP Hippocampus	ALL	2way ANOVA	Interaction: *<br>Age: ns<br>Diet: ns	Interaction: 0.0164<br>Age: 0.3531<br>Diet: 0.1239	Interaction: $F_{(3,29)}$ = 4.031<br>Age: $F_{(3,29)}$ = 1.130<br>Diet: $F_{(1,29)}$ = 2.512
Fig.4. APP Hippocampus	4M CTR vs. 4M WD	Tukey's post-hoc	*	0.0424	-

*p<0.05, ****p<0.0001

Table S4: Completed statistical data – comparable analysis of brain markers in Tg2576 (APPswe) mice (paragraph 2.4)

Figure number: Analyzed parameter	Age groups	Statistical test	Significance	p-Value	Value of F (DFn, DFd)
Fig.6A. p-IRS-1 (Ser616) Entorhinal cortex	ALL	2way ANOVA	Interaction: ns<br>Age: **<br>Diet: *	Interaction: 0.3039<br>Age: 0.0050<br>Diet: 0.0122	Interaction: $F_{(2,19)}$ = 1.269<br>Age: $F_{(2,19)}$ = 7.094<br>Diet: $F_{(1,19)}$ = 7.663
Fig.6A. p-Tau (Thr231) Entorhinal cortex	ALL	2way ANOVA	Interaction: ns<br>Age: ***<br>Diet: **	Interaction: 0.1109<br>Age: 0.0004<br>Diet: 0.0032	Interaction: $F_{(2,19)}$ = 2.474<br>Age: $F_{(2,19)}$ = 12.31<br>Diet: $F_{(1,19)}$ = 11.35
Fig.6A. p-Tau (Thr231) Entorhinal cortex	4M vs. 4M CTR WD	Tukey's post-hoc	*	0.0284	-
Fig.6A. p-Tau (Thr231) Entorhinal cortex	4M vs. 8M CTR CTR	Tukey's post-hoc	**	0.0048	-
Fig.6A. p-Tau (Thr231) Entorhinal cortex	4M vs. 12M CTR CTR	Tukey's post-hoc	*	0.0194	-
Fig.6A. APP Entorhinal cortex	ALL	2way ANOVA	Interaction: ns<br>Age: ns<br>Diet: ns	Interaction: 0.4321<br>Age: 0.1534<br>Diet: 0.2377	Interaction: $F_{(2,19)}$ = 0.8774<br>Age: $F_{(2,19)}$ = 2.073<br>Diet: $F_{(1,19)}$ = 1.486
Fig.6B. p-IRS-1 (Ser616) Hippocampus	ALL	2way ANOVA	Interaction: ns<br>Age: ns<br>Diet: ns	Interaction: 0.3754<br>Age: 0.0504<br>Diet: 0.2780	Interaction: $F_{(2,21)}$ = 1.027<br>Age: $F_{(2,21)}$ = 3.456<br>Diet: $F_{(1,21)}$ = 1.240
Fig.6B. p-Tau (Thr231) Hippocampus	ALL	2way ANOVA	Interaction: ns<br>Age: ns<br>Diet: ns	Interaction: 0.1660<br>Age: 0.9990<br>Diet: 0.6162	Interaction: $F_{(2,19)}$ = 1.977<br>Age: $F_{(2,19)}$ = 0.001<br>Diet: $F_{(1,19)}$ = 0.26
Fig.6B. APP Hippocampus	ALL	2way ANOVA	Interaction: *<br>Age: ***<br>Diet: ns	Interaction: 0.0272<br>Age: 0.0009<br>Diet: 0.0710	Interaction: $F_{(2,20)}$ = 4.338<br>Age: $F_{(2,20)}$ = 10.24<br>Diet: $F_{(1,20)}$ = 3.635
Fig.6B. APP Hippocampus	4M vs. 4M CTR WD	Tukey's post-hoc	*	0.0277	-
Fig.6B. APP Hippocampus	4M vs. 8M CTR CTR	Tukey's post-hoc	**	0.0059	-
Fig.6B. APP Hippocampus	4M vs. 12M CTR CTR	Tukey's post-hoc	**	0.0018	-

*p<0.05, **p<0.01
